# A surface registration‐based approach for assessment of 3D angles in guided growth interventions in the growing femur

**DOI:** 10.1002/jeo2.12111

**Published:** 2024-07-29

**Authors:** Shima Gholinezhad, John Rasmussen, Ahmed Halloum, Søren Kold, Ole Rahbek

**Affiliations:** ^1^ Department of Orthopedic Surgery Aalborg University Hospital Aalborg Denmark; ^2^ Department of Medicine Aalborg University Aalborg Denmark; ^3^ Department of Materials and Production Aalborg University Aalborg Denmark

**Keywords:** 3D imaging, femoral rotation, guided growth, postoperative assessment

## Abstract

**Purpose:**

Postoperative assessment of surgical interventions for correcting femoral rotational deformities necessitates a comparative analysis of femoral rotation pre‐ and post‐surgery. While 2D assessment methods are commonly employed, ongoing debate surrounds their accuracy and reliability. To address the limitations associated with 2D analysis, we introduced and validated a 3D model‐based analysis method for quantifying the angular and rotational impact of corrective rotational osteotomy in the growing femur.

**Methods:**

The method is based on surface registration of the pre‐ and post‐intervention 3D femoral models. To this end, 3D triangulated surface models were generated using CT images for the right femurs of 11 skeletally immature pigs, each scanned at two distinct time points with a 12‐week interval between scans. In our validation procedures, femoral corrective rotational osteotomy of the post‐12‐week femur was simulated at varying angles of 5, 10, 15 and 20 degrees in three dimensions. Subsequently, a surface 3D/3D registration‐based approach was applied to determine the 3D femoral angulation and rotation between the two models to assess the method's detection accuracy of the predefined twist angles as ground truth references.

**Results:**

The results document the precision and accuracy of the registration‐based method in evaluating rotation angles. Consistently high accuracy was observed across all angles, with an accuracy rate of 92.97% and a coefficient of variance of 8.14%.

**Conclusion:**

This study has showcased the potential for improving post‐operative assessments with significant implications for experimental studies evaluating the effects of correcting rotational deformities in the growing femur.

**Level of Evidence:**

Not applicable.

**Abbreviations**
2D: two‐dimensional
3D: three‐dimensional
ICP: Iterative Closest Point
W0: Week 0
W12: Week 12
ΔFR: femoral rotation pre‐ versus postsurgery

## INTRODUCTION

Rotational deformity of the femur might result in pain and altered gait, such as in‐toeing or out‐toeing. Traditionally, femoral rotational deformity has been treated surgically by corrective osteotomy, where the femoral bone is de‐rotated along its long axis either gradually or acutely [23]. Recently, correction of femoral rotational deformity by use of guided growth has been proposed as an option in growing children [37]. However, the impact of such new procedures must be thoroughly examined prior to introduction into clinical use.

**Figure 1:**
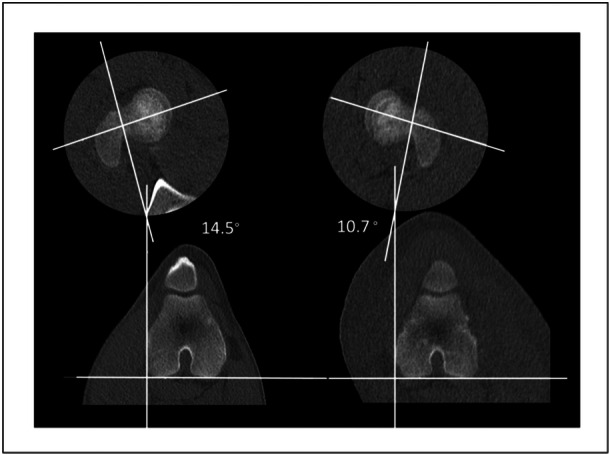
Femoral rotation for the right and left sides in one of the pig models was measured as the difference between the femoral neck axis (determined using the Lee method [19]) and the tangent line drawn across the axis of the posterior femoral condyles.

Evaluation of orthopaedic surgical outcomes typically relies on two‐dimensional (2D) slice‐based CT scans taken at a minimum of two time points, serving as the gold standard [10, 19, 22, 26, 33]. As shown in Figure 1, femoral torsion at each time point is measured using axial cuts, quantifying the relative angle between the long axis of the femoral neck and the posterior apex of the distal femoral condyles [19]. The comparative analysis between the time points documents the extent of femoral rotation (FR) pre‐ versus post‐surgery (hereafter denoted ΔFR).

Despite its widespread use in orthopaedics, the 2D analysis approaches possess inherent limitations when it comes to precisely evaluating complex rotational deformities in long bones. First, uncertainty arises when selecting the appropriate 2D slices on axial CT images to define the landmarks [38]. Furthermore, the subjective choice of bone landmarks, specifically when selecting the proximal femoral reference axis depending on the measurement technique, may lead to several examples of contradictory findings [14, 20, 28, 31, 32]. This issue may be exacerbated in animal experimental studies, such as those using porcine models, where the short femoral neck adds to the difficulty of consistently identifying the proximal landmark (Figure 1).

These limitations have led to a growing emphasis on post‐operative orthopaedic assessments using 3D analysis, with studies measuring femoral torsion through three‐dimensional (3D) femur shape reconstructions from various imaging modalities such as CT [25], MRI [9] and EOS scans [21]. In these studies, a landmark‐based method is commonly applied, which involves measuring the femoral torsion as the angle between the femoral neck axis and the retrocondylar plane. Nevertheless, consistent reliability in identifying these landmarks may remain a challenge [8], also relying solely on a single set of landmark points may not adequately account for rotational changes in both the coronal and sagittal planes as side effects of the intervention [7, 15, 18, 24].

There is a proposal to employ the surface registration of the two pre‐ and post‐operative 3D femur models for evaluating the ΔFR [1]. In this approach, the surfaces of the two models are superimposed to identify discrepancies across all *x, y* and *z* coordinates, representing changes between the pre‐ and post‐treatment femur models. The benefit of surface registration is its ability to assess deformities in all anatomical planes, as highlighted previously [35]. However, surface registration has a finite and unknown accuracy and may be prone to errors from mesh irregularities and medical image distortion. Therefore, in the current study, we perform a systematic verification and validation of angle detection by a surface registration‐based approach. The concept of surface registration was applied to evaluate ΔFR by aligning pre‐ and post‐intervention femur models. Specifically, we reconstructed 3D femoral models using CT images from skeletally immature growing porcinis without pathological conditions, separated by a 12‐week interval between scans. The surgical intervention was simulated by artificially twisting the femur models at known angles. The proposed algorithm was then tasked with detecting these predetermined angles, serving as a validation against the ground truth values. The degree to which the algorithm can accurately detect the rotational angles served as an indicator of its accuracy.

## MATERIALS AND METHODS

This study obtained CT scans of the right femur from 11 skeletally immature pigs without femoral pathology at two distinct time points. The initial scans were conducted when the pigs were approximately three months old, denoted as Week 0 (W0) scans. A second set of scans was performed 12 weeks later, referred to as Week 12 (W12) scans. The full length of the femur was available for all 11 pigs. The scans were conducted using a CT Canon Aquilion Prime SP (Canon Medical Systems). The CT scans had a resolution of 512 × 512 pixels, and the slice thickness was 0.8 mm in all scans. The mean weight of the pigs was 42 kg (range 38–45 kg) and 84 kg (range 80–94 kg) in W0 and W12, respectively. The study was approved by the Danish Animal Experiments Inspectorate under the application number 2020‐15‐0201‐00690.

The CT scans were segmented using the global thresholding and region‐growing functions of standard segmentation software (Mimics Medical, Materialise NV). The segmentation resulted in the generation of 3D triangulated mesh bone models [3], which were then imported into Python for further analysis using the Trimesh library [6].

**Figure 2:**
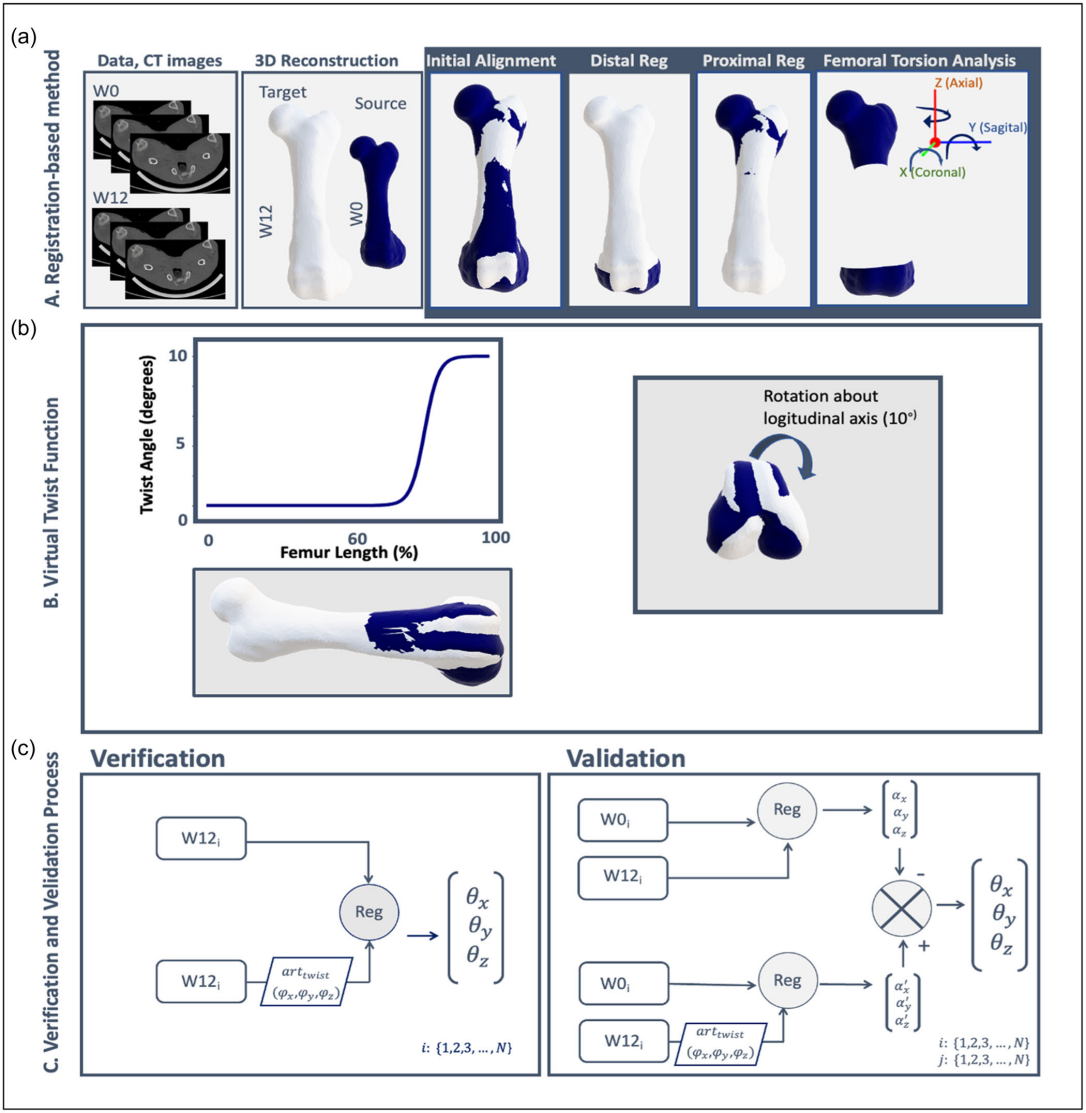
Panel (a) illustrates the registration‐based method. 3D femurs were generated using computed tomography scan data obtained from the right femur at two distinct time points. In the first step, the 3D reconstruction of the W0 scan was aligned with the W12 scan using Iterative Closest Point (ICP) (initial alignment). The W0^T^ was then sectioned into proximal and distal segments. Next, the ICP algorithm was employed to align the proximal and distal segments of W0^T^ with its counterpart in W12. The disparity in orientation between the proximal and distal sections of the W0^T^ before and after registration to the W12 reflects the femoral rotation difference between W0 and W12 femur models. The virtual twist function (b) is employed to artificially rotate the femoral model (depicted in white) around the *X*, *Y* and *Z* axes. In this figure, the twist is illustrated around the *Z*‐axis and the resulting rotated bone is shown in navy. Verification and validation processes (c) involve calculating the ΔFR between W12 and artificially twisted W12 at known angles within the same bone (left panel) to verify the accuracy and functionality of the computer algorithm. Validation (right panel) involves aligning W12 femur models from one pig with W0 models from the remaining pigs to assess the method's performance in handling diverse bone structures.

To quantify the ΔFR, we utilized a 3D registration‐based approach, as depicted in Figure 2a. The initial step in this approach centres on the preliminary alignment, incorporating scaling, rotation and translation operations to register the W0 on top of the W12 model while preserving its shape. This is necessary to account for the variations in size and spatial position between the W0 and W12 meshes occurring due to femoral growth and placement uncertainty in the scanner. This is achieved by the Iterative Closest Point (ICP) algorithm [1, 2], which minimizes disparities between the two models by optimizing scaling and translation parameters, ensuring the best fit between the W0 and W12 femurs. The result is referred to as W0^T^. In the interest of the robustness of the ICP registration, it was proceeded by preregistration of the two bones by alignment of their principal inertial axes.

Next, the ΔFR over 12 weeks is determined as the disparity in orientation between the proximal and distal sections of the W0^T^ before and after their sectioning and respective registration to the W12, as illustrated in Figure 2a. Specifically, the proximal section comprises 40% of the bone length, including the femoral head, femoral neck, and both trochanters and a portion of the shaft, and the distal section encompasses 15% from the distal end, including the condyles. The ΔFR is characterized as a 3D vector of Euler angles or a rotation vector. A 3D ΔFR of (0, 0, 0) degrees signifies no rotational differences between the models captured at two different time points. While this approach captures the total rotation between the two selected sections between the two time points, studies suggest that torsional differences may not be homogeneously distributed over the entire femur in human models [11]. Therefore, different sectioning methods may be required to assess torsional differences relative to the pathological area.

The performance of the approach was verified and validated against a contrived ground truth reference. The ground truth was established by simulating distal femoral rotation in all three planes. The applied twist function operates by rotating the femur about the *X*, *Y* and *Z* axes along defined twisting regions located 40% of the length from the distal end. This ensures a smooth and gradual increment in the twist angle extending towards the distal end (Figure 2b). We specifically rotated the bone at angles of 5, 10, 15 and 20 degrees. The incorporation of four angles aimed to comprehensively test the algorithm across varying degrees of rotation, encompassing both small and large ranges.

As shown in Figure 2c, to verify the algorithm, we applied the method to determine the ΔFR between each of the W12 femurs and their artificially twisted counterparts (Figure 2c, left panel). In the next validation procedure, the method was tested in each of the eleven pigs by determining the rotation angles between the W0 femur and the twisted W12 femur. To account for potential femoral rotation naturally occurring due to growth effects, the method was first applied on the same investigating pairs without twisting the W12 femurs. The ΔFR was then calculated by accounting for rotation angles influenced by growth‐related effects and subtracting them from the angles obtained during the application of virtual twisting for each corresponding pair. For validation, 484 (11 × 11 × 4) registration experiments were conducted, applying the four twist angles (true values) across all eleven femurs. In both verification and validation, a closer alignment between the detected ΔFR and the applied twisting angle indicates better performance.

*Result visualization* **:** To visualize the detected angles by the algorithm with ground truth values, we used Bland–Altman plots, which illustrate the accuracy dependency on nominal true values. The detected angles were also expressed as a percentage of the true values. The average of normalized detected angles for each of the three axes (*X, Y, Z*) and the four twist angles (5, 10, 15 and 20 degrees) across all registration experiments for both validation and verification were then computed. We refer to the absolute error between the average normalized detected angles and the true angles (i.e., 100%) as the detection error. Additionally, the coefficient of variance of the measurements indicating the degree of dispersion of detected angles around the true angle (i.e., 100%) was computed.

## RESULTS

**Figure 3:**
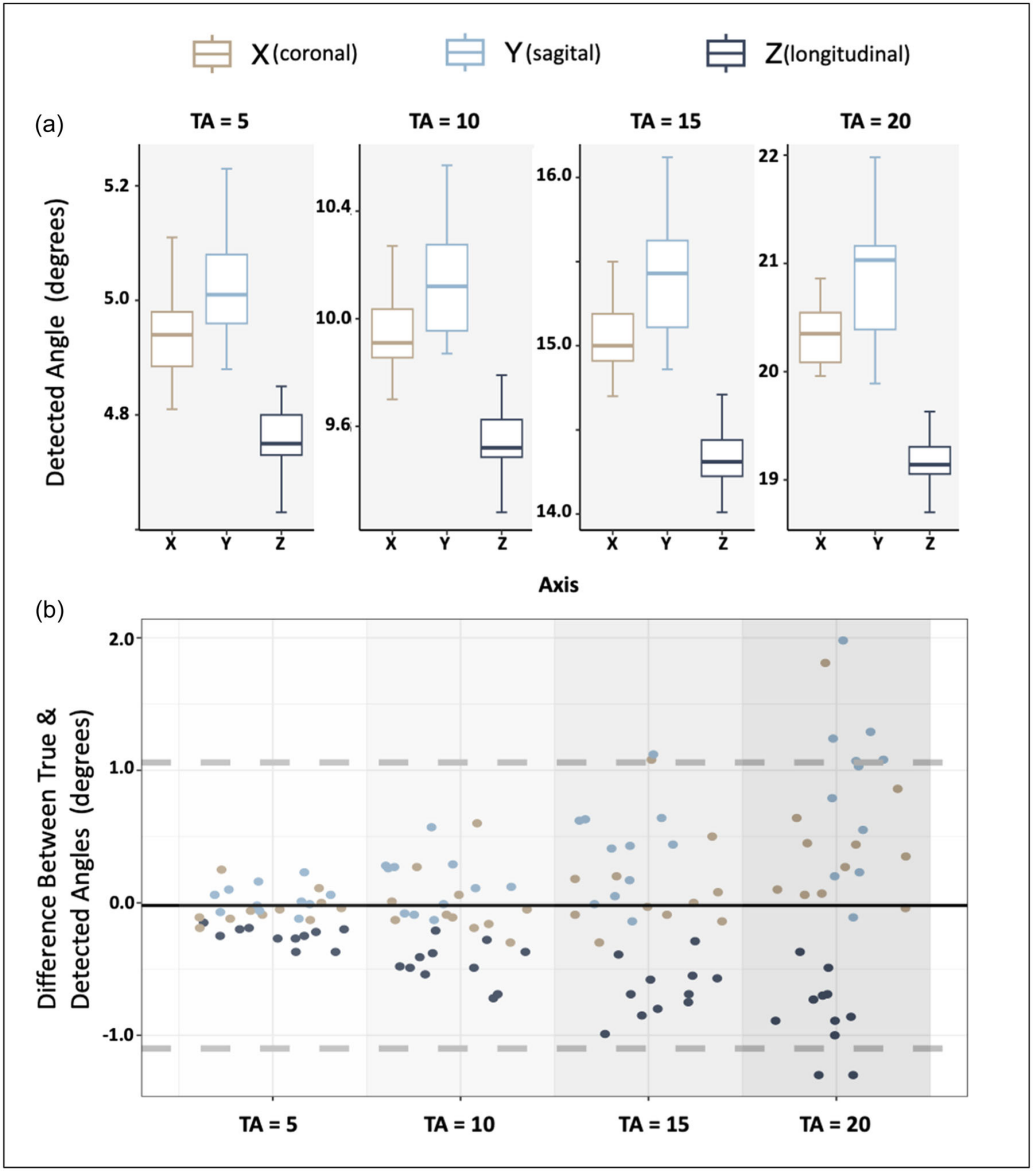
Verification results: Box plots showcasing detected angles (a) across all 11 subjects displayed for each of the three dimensions, with different true angles highlighted in each facet. In the Bland–Altman figure (b), the vertical axis represents the relative difference between true and detected angles, and the horizontal axis represents the true angles. The figure incorporates all cases across all axes, with X depicted in beige, Y in blue and Z in navy. The black line signifies the mean, while the dashed grey lines indicate the upper and lower limits of agreement.

The outcome from verification indicated that the detected angles approximated the true angles with high accuracy across all cases. Figure 3a shows that for the X dimension, the detected twist angles were approximately 4.96° ± 0.12° for a true angle of 5°, 9.99° ± 0.25° for 10°, 15.12° ± 0.38° for 15° and 20.45° ± 0.52° for 20°. For the Y dimension, the corresponding detected angles were 5.03° ± 0.10°, 10.14° ± 0.21°, 15.39° ± 0.36° and 20.85° ± 0.59°. In the Z dimension, the detected angles were 4.75° ± 0.07°, 9.54° ± 0.15°, 14.35° ± 0.20° and 19.26° ± 0.29°. The Bland–Altman plot in Figure 3b shows a mean error of zero between true and detected angles, with limits of agreement from −1.09 to 1.05. The algorithm tends to underestimate *Z*‐axis rotation and overestimate *Y*‐axis rotation, indicating minor interference between dimensions.

**Figure 4:**
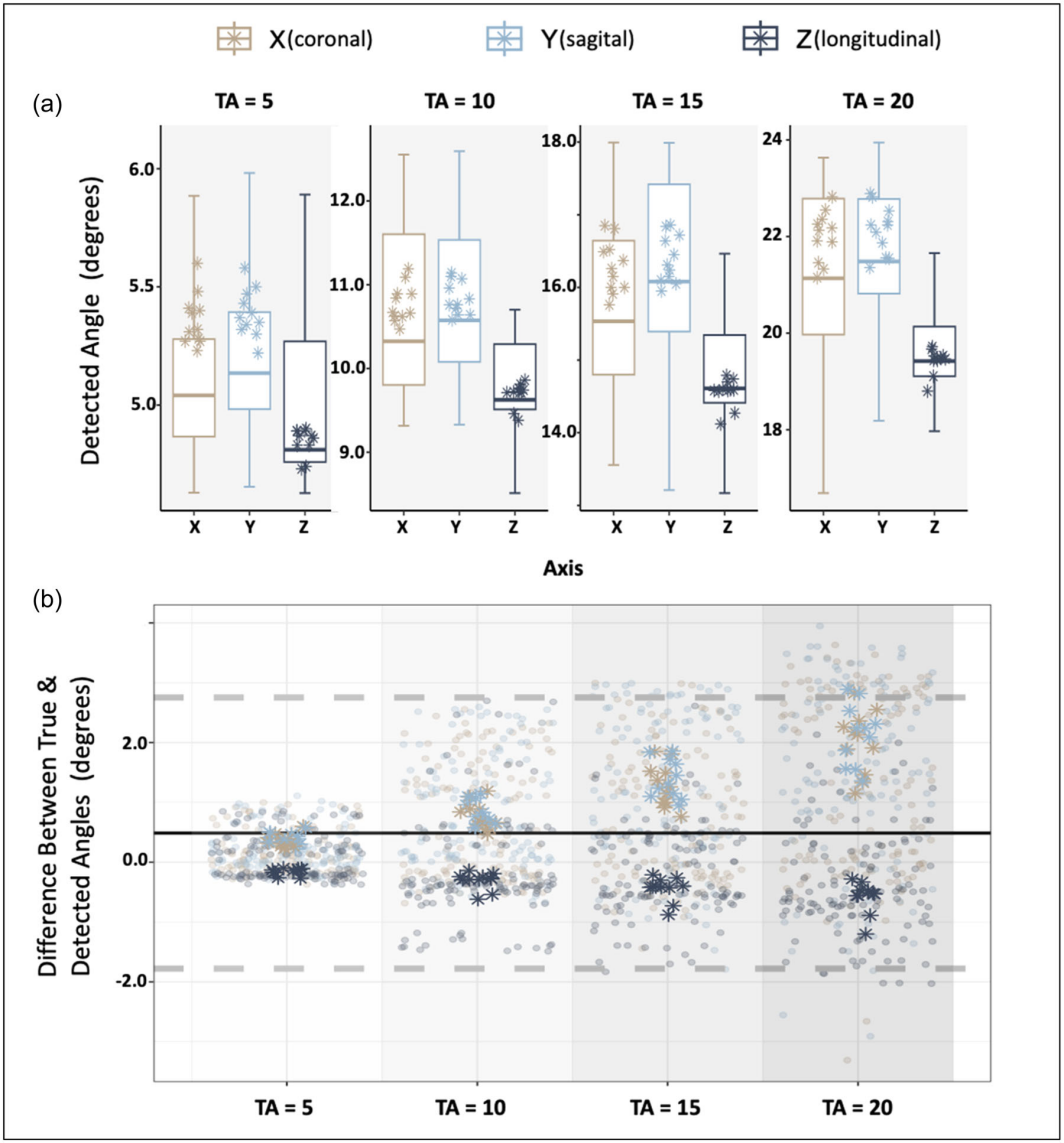
Validation results: Box plots showcasing detected angles (a) across all 11 subjects displayed for each of the three dimensions, with different true angles highlighted in each facet. In the Bland–Altman (b), the vertical axis represents the difference between true and detected angles, and the horizontal axis represents the true angle. The figure incorporates all cases across all axes, with X depicted in beige, Y in blue and Z in navy. The black line signifies the mean, while the dashed grey lines indicate the upper and lower limits of agreement. Star points indicate the outcome of the registration experiment when the W0 and W12 scans of the same pig were applied.

The validation process involved bones from different time points (W0 and W12) and different femur models. As shown in Figure 4a for the corresponding true angle of 5°, 10°, 15° and 20°, the detected angles were 5.13° ± 0.34°, 10.69° ± 0.99°, 15.76° ± 1.12° and 21.26° ± 1.52° for the X dimension, 5.21° ± 0.34°, 10.82° ± 0.95°, 16.19° ± 1.23°, 21.46° ± 1.51° for the Y dimension and 4.96° ± 0.80°, 9.83° ± 0.85°, 14.80° ± 0.75°, 19.60° ± 0.89° for the Z dimension. Similar to verification, we observed an underestimation of the detected angle in the *Z*‐axis and an overestimation in the *X*‐ and Y‐axis. As indicated by Figure 4b, a mean error of 0.48° was found between the detected and true angles. The limits of agreement (LoA) were −1.77 and 2.75, indicating the spread of differences across dimensions and true angles. Despite larger errors with larger true angles, the consistent error percentage suggests steady relative accuracy across different true angles.

**Figure 5:**
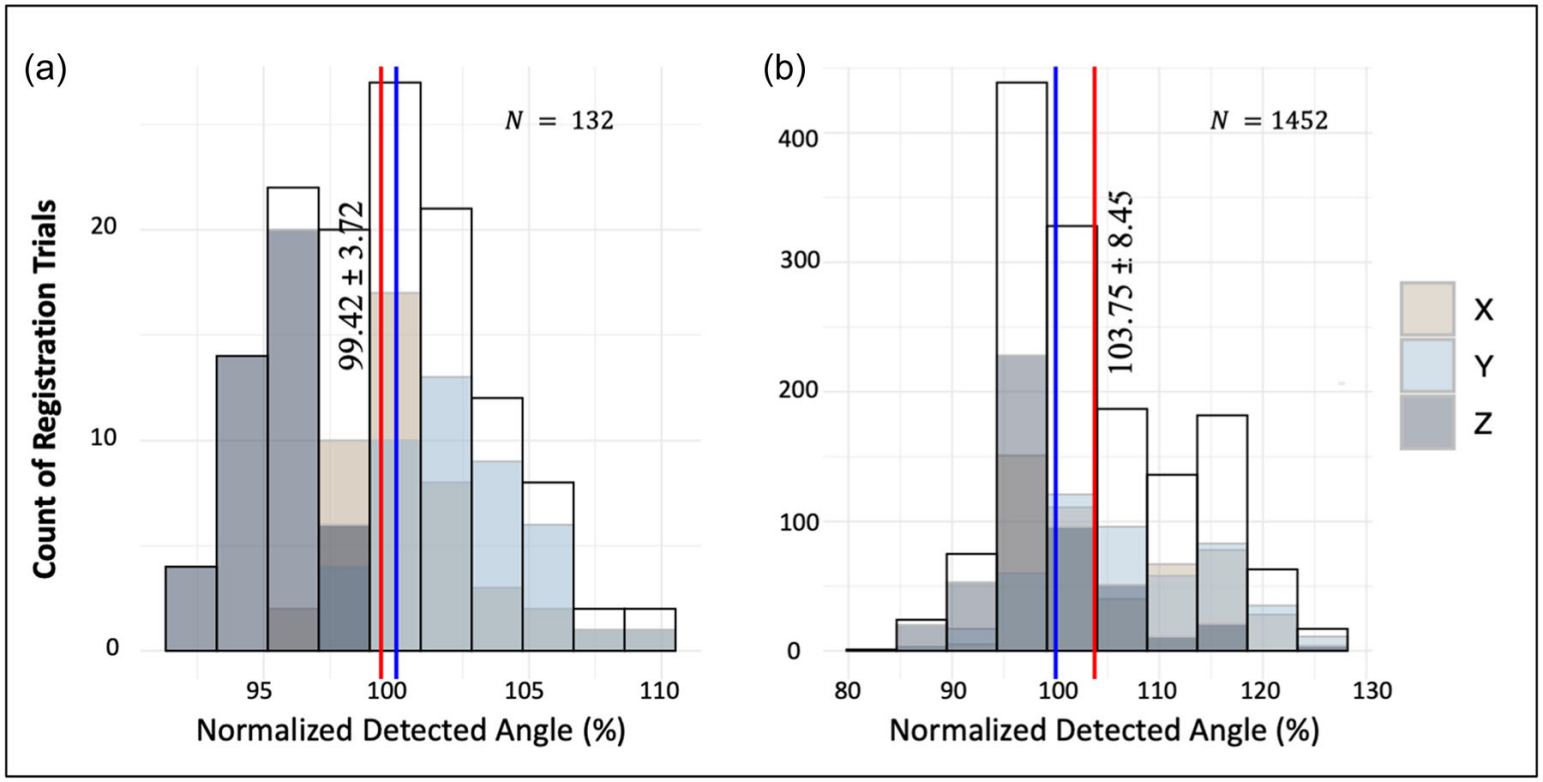
Distribution of detected angles normalized to the respective true angle across all rotation axes and registration trials for the verification (a) and validation (b) procedures. The red, vertical line indicates the mean of normalized detected angles in each distribution. And the blue lines indicate the true angle (i.e., 100%).

Throughout all registration trials, the mean of the normalized detected angles was determined to be 99.42 ± 3.72% for the verification process and 103.75 ± 8.45% for validation (Figure 5). Across all true angles and dimensions, using the normalized detection angles, the associated mean absolute error values were calculated as 3.06 ± 2.17% for verification and 7.02 ± 6.01% for validation.

## DISCUSSION

The aim of this study was to evaluate the accuracy of the registration‐based approach in measuring ΔFR in growing femurs by comparing it against ground truth values. To accomplish this, controlled scenarios involving virtual bone twisting about the anatomical axis at certain angle values were implemented and validated. The mean absolute error was 3.06 ± 2.17% for verification and 7.02 ± 6.01% for validation. These numbers indicate an acceptable accuracy and consistency in detecting changes in rotational angles, regardless of morphological changes in the shape of the femur before and after surgery.

In the literature, the assessment of femoral torsion using 3D femur models often relies on landmark registration. In such approaches, proximal and distal femoral 3D models are realigned to the mechanical axis, and femoral torsion is evaluated as the intersection between the femoral neck axis and the retrocondylar plane [4, 5, 8, 11]. However, it has been demonstrated that 3D measurements can be sensitive to how the femoral neck axis is defined [8]. In this recent study, van Fraeyenhove et al. conducted a study examining femoral torsion through five different 3D analyses based on the selection of the femoral neck axis. Their findings revealed significant variations in results depending on how the femoral neck axis was defined (*p* < 0.001). In contrast, the registration‐based method gathers high‐dimensional landmark data and automatically extracts 3D surface information, which may reduce the impact of intra‐rater bias in measurements.

The approach applied in this study offers an advantage over other 3D femoral torsion assessors in its capacity to evaluate femoral rotation in three dimensions, these methods facilitate the three‐dimensional (3D) evaluation of deviations from the pre‐intervention model across all anatomical planes and enable 3D post‐operative assessment. In many proposed 3D assessment studies, the femoral rotation is evaluated in the transverse plane only. This approach may not be optimal for assessing derotational corrections, including torsional osteotomies [30] and growth modulation [37], as unforeseen effects on the remaining planes and adjacent joints have been observed clinically [13, 16, 18, 24]. For instance, it has been shown that proximal and distal femoral derotational osteotomies may impact frontal plane alignment, resulting in varus and valgus effects, respectively [24]. Therefore, 3D post‐operative evaluation quantifying 3D ΔFR is relevant specifically when introducing novel surgical approaches [17].

The validation process applied in the current study also differs from similar research. Here, the accuracy of 3D model analysis for femoral torsion was directly evaluated against ground truth measurements. In this way, we used synthetic validation scenarios to establish an accurate and reliable validation method. This approach differs from the evaluation strategies used in studies advocating for 3D femoral analysis. In such studies, the effectiveness of a novel 3D femoral rotation assessor is juxtaposed with conventional 2D analysis [4, 5, 8, 12, 25]. For example, in Iwasaka–Neder et al. [12], the reliability of the assessment of femoral torsion through a 3D femur model is compared to Murphy's 2D axial technique [22]. Another study by Brooks et al. [4] introduced a 3D analysis for measuring the femoral version, and they compared their approach to the 2D technique described by Reikerås et al. [26]. Contrasting 3D approaches with different 2D methods may pose a controversy, given the substantial variations in reported values for femoral torsion observed across different 2D measurement methodologies in different studies [28, 29]. For instance, in Schmaranzer et al. [28], the femoral torsion values assessed by Reikerås et al. (15.2° ± 14.2°) significantly differ from those by Murphy et al. (29.0° ± 14.5°) (*p* < 0.0001 [28]). While the comparison of result variability between 2D and 3D analyses may emphasize the consistency of 3D analysis, it is crucial to note the challenges in determining the accuracy of 3D analysis. This limitation stems from the lack of a valid reference for thorough validation.

Diverging from the conventional practices commonly observed in femoral rotation assessments (either 2D or 3D analysis), which typically involve measurements separately on individual pre‐ and post‐intervention models, followed by calculating the difference between them, our approach involves directly calculating the change in femoral rotation (ΔFR) through the fusion of femoral models. This concept has previously been applied in the preplanning phase of surgeries, where a model of the pathological side is superimposed onto the normal anatomy (ipsilateral vs. contralateral or statistical template [16, 27, 34, 35]) through surface registration. In these studies, the evaluation of the 3D rotational difference was aimed at correcting the pathological femur. It is important to highlight that, unlike those studies, the current study compensates for a size difference between the two models due to the element of substantial growth occurring over the 12‐week period, which results in varying sizes and shapes between the pre‐ and post‐intervention models. This was done to investigate whether the presence of a growth factor, indicating changes in the size and shape of the femur, might impact the algorithm's ability to detect the effects of rotational corrective procedures.

The limitations of the study are primarily associated with the manual segmentation process. First, in terms of processing time, the manual segmentation of the femur may require up to 2 h and may be further complicated by the removal of metal artefacts, particularly in cases involving implants. Automated algorithms [36, 39, 40] promise to improve this situation. Another significant limitation is the inherent interdependence between registration‐based methods and the accuracy of the segmentation process. The manual segmentation and resampling of images at various stages in the pipeline have the potential to impact the reproducibility of results. To address this concern, future studies should consider investigating intra‐ and interobserver bias associated with the manual approach.

In conclusion, the outcomes of this study underscore the precision and accuracy of the 3D analysis based on surface registration of the pre‐ and post‐treatment femur models in evaluating the angular and rotational impacts of the corrective rotational osteotomy. This approach may have significant implications for studies assessing the effects of correcting rotational deformities in the growing femur.

## AUTHOR CONTRIBUTIONS

*Study conception and design*: All authors. *Data acquisition*: Ahmed Halloum. *Methodology development and implementation*: John Rasmussen and Shima Gholinezhad. *Analysis*: Shima Gholinezhad. *Interpretation of results*: John Rasmussen and Shima Gholinezhad. *Draft manuscript preparation*: Shima Gholinezhad. All authors reviewed the results and approved the final version of the manuscript.

## CONFLICT OF INTEREST STATEMENT

The authors declare no conflict of interest.

## ETHICS STATEMENT

The study was approved by the Danish Animal Experiments Inspectorate under the application number 2020‐15‐0201‐00690.

## Data Availability

The datasets used and/or analysed during the current study are available from the corresponding author upon reasonable request.
